# Correction: Kalvala et al. Cannabidiol-Loaded Extracellular Vesicles from Human Umbilical Cord Mesenchymal Stem Cells Alleviate Paclitaxel-Induced Peripheral Neuropathy. *Pharmaceutics* 2023, *15*, 554

**DOI:** 10.3390/pharmaceutics15092200

**Published:** 2023-08-25

**Authors:** Anil Kumar Kalvala, Arvind Bagde, Peggy Arthur, Tanmay Kulkarni, Santanu Bhattacharya, Sunil Surapaneni, Nil Kumar Patel, Ramesh Nimma, Aragaw Gebeyehu, Nagavendra Kommineni, Yan Li, David G. Meckes, Li Sun, Bipika Banjara, Keb Mosley-Kellum, Thanh Cong Dinh, Mandip Singh

**Affiliations:** 1 Department of Pharmaceutics, College of Pharmacy and Pharmaceutical Sciences, Florida A&M University, Tallahassee, FL 32301, USA; 2 Department of Biochemistry and Molecular Biology, Mayo College of Medicine and Science, Jacksonville, FL 32224, USA; 3 Department of Physiology and Biomedical Engineering, Mayo College of Medicine and Science, Jacksonville, FL 32224, USA; 4 College of Engineering, Florida A&M University-Florida State University, 2525 Pottsdamer St., Tallahassee, FL 32310, USA; 5 Department of Biomedical Sciences, Florida State University College of Medicine, 1115 West Call Street, Tallahassee, FL 32301, USA

“Yan Li” was not included as an author in the original publication [1]. Dr. Yan Li designed, performed, and wrote up the immunocytochemistry experiment and also revised the manuscript. The corrected Author Contributions statement appears here:

**Author Contributions:** A.K.K. conceptualized, designed, performed, and analyzed the results and wrote the manuscript. P.A., A.B., T.K., S.B., S.S., N.K.P., R.N., A.G., N.K., D.G.M.J., L.S., B.B., K.M.-K. and T.C.D. performed part of the experimental work and/or analyzed the results T.K., designed, performed, analyzed and wrote AFM experiments. S.B. conceptualized and revised AFM experimental outcome. M.S. conceptualized the study, wrote, and revised the manuscript. Y.L. designed, performed, and wrote up the immunocytochemistry experiment and also revised the manuscript. All authors have read and agreed to the published version of the manuscript.

The authors state that the scientific conclusions are unaffected. This correction was approved by the Academic Editor. The original publication has also been updated.
