# Supplementary material for: Cannabidiol-Loaded Extracellular Vesicles from Human Umbilical Cord Mesenchymal Stem Cells Alleviate Paclitaxel-Induced Peripheral Neuropathy
Source: Pharmaceutics. 2023 Feb 7;15(2):554. doi: 10.3390/pharmaceutics15020554 (PMC9964872; doi:10.3390/pharmaceutics15020554)

Cannabidiol Loaded Extracellular Vesicles from Human Umbilical Cord Mesenchymal Stem Cells Alleviate Paclitaxel Induced Peripheral Neuropathy

Anil Kumar Kalvala[a], Peggy Arthur[a], Arvind Badge[a], Tanmay Kulkarni[b], Santanu Bhattacharya[bc], Sunil Surapaneni[a], Nil Kumar Patel[a], Nimma Ramesh[a], Aragaw Gebeyehu[a], Nagavendra Kommineni[a], David G Meckes Jr[d], Li Sun[d], Bipika Banjara[a], Keb Mosley[a], Thanh Cong Dinh[a], Mandip Singh[a@]

a.College of Pharmacy and Pharmaceutical Sciences, Florida A&M University, Tallahassee, Florida, 32307, USA.

b.Department of Biochemistry and Molecular Biology, Mayo College of Medicine and Science, Jacksonville, FL, USA.

c.Department of Physiology and Biomedical Engineering, Mayo College of Medicine and Science, Jacksonville, FL, USA.

d. Department of Biomedical Sciences, Florida State University College of Medicine, 1115 West Call Street, Tallahassee, FL, 32306-4300, USA

Abbreviated Title: Role of CBD (MSC)-derived EVs formulation in experimental Paclitaxel Induced Neuropathy

Funding source: Consortium for Medical Marijuana Clinical Outcomes Research, Grant/Award number: SUB00002097, **National Institute on Minority Health and Health Disparities of National Institutes of Health**, Grant/Award Number: U54 MD007582 and **NSF-CREST Center for Complex Materials Design for Multidimensional Additive Processing (CoManD)**, Grant/Award Number:1735968

@Correspondence
Mandip Singh, Professor and Section Leader, Pharmaceutics,
College of Pharmacy and Pharmaceutical Sciences,
Florida A&M University
Tallahassee, FL 32307
Phone: 850-561-2790, Fax:850-599-3813
E-mail: mandip.sachdeva@famu.edu, mandip.sachdeva@gmail.com

1. Supplementary methods

1.1 Chemicals and antibodies:

Table S1: materials used in the present study with the source and catalog number

Sr. No	Chemical name	Key source	CAT#Number
1	Cannabidiol (GLP and GMP grade)	PurisysTM (Athens, GA)	NQS1951
2	Paclitaxel	LC Laboratories (MA, USA)	P-9600
3	α-MEM media	Millipore Sigma (St. Louis, MO)	M4526
4	Fetal bovine serum (FBS)	Atlanta biological, MN, USA	S11150
5	Human umbilical cord derived mesenchymal stem/stromal cells	Dr. David Meckes, FSU College of Medicine Florida State University	
6	PBS-vertical wheel bioreactor	PBS Biotech, Inc (Camarillo, CA).	200051BLEM
7	Cytodex-1 microcarrier	VWR International (Radnor, PA),	17-0448-03
8	NADH Assay Kit	Millipore Sigma (St. Louis, MO)	MAK037
9	ATP Assay Kit	Millipore Sigma (St. Louis, MO)	MAK190
10	P-AMPK alpha antibody	Cell signalling technology, USA	2535S
11	Sirt1 antibody	Cell signalling technology, USA	9475S
12	NRF2 antibody	Cell signalling technology, USA	12721S
13	NRF1 antibody	Cell signalling technology, USA	46743S
14	TFAM antibody	Santacruz biotechnologies, CA	166965
15	PINK 1 antibody	Santacruz biotechnologies, CA	517353
16	HO-1 antibody	Cell signalling technology, USA	43966S
17	SOD2 antibody	Cell signalling technology, USA	13141S

18	Catalase antibody	Santacruz biotechnologies, CA	271803
19	CB1 antibody	Santacruz biotechnologies, CA	J1920
20	5HT1A antibody	Abcam, MA, USA	85615
21	β-Actin antibody	Cell signalling technology, USA	4970S
22	Parkin Antibody	Santacruz biotechnologies, CA	32282
23	SIRT3 antibody	Santacruz biotechnologies, CA	365175
24	Mouse anti-rabbit IgG-CFL 488	Santacruz biotechnologies, CA	516248
25	Rabbit anti-mouse IgG-PE	Santacruz biotechnologies, CA	358926
26	Flotillin-2	Santacruz biotechnologies, CA	28320
27	CD 63	Santacruz biotechnologies, CA	365604
28	CD81	Santacruz biotechnologies, CA	166029
29	Alix	Santacruz biotechnologies, CA	53538
30	Calnexin	Santacruz biotechnologies, CA	23954

Figure S1. Effect of CBD and CBD-EVs on neuritogenesis in cultured DRG cells isolated from L1 to L5 spinal region of Rats. (a) Representative photomicrographs captured by using phase contrast microscope (40X) showing the neurite extensions coming out from each DRG cell, (b) bar graphs represents the percent of cells bearing neurites and (c) bar graph represent the image j analysis of neurite length of untreated and treated groups. ***p<0.001 Vs NC, ^p<0.05, ^^p<0.01 and ^^^p<0.001 Vs PTX (8 mg/kg), $P<0.05 and $$P<0.01 Vs PTX+CBD.

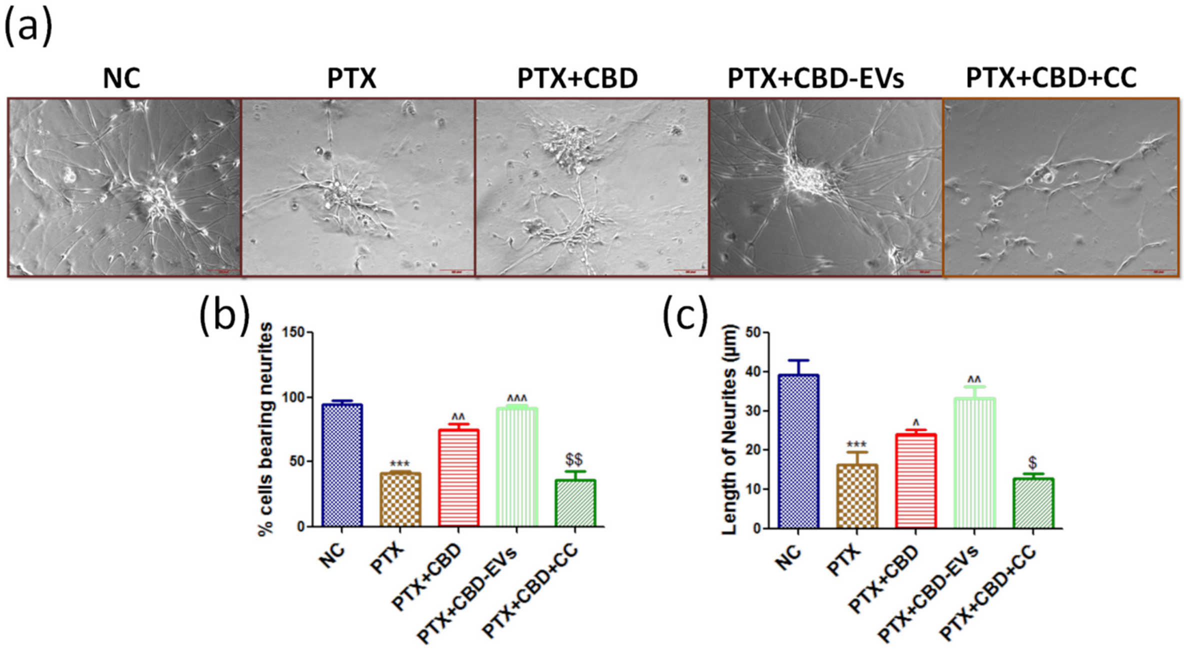

Figure S2: Raw blots of all the markers:

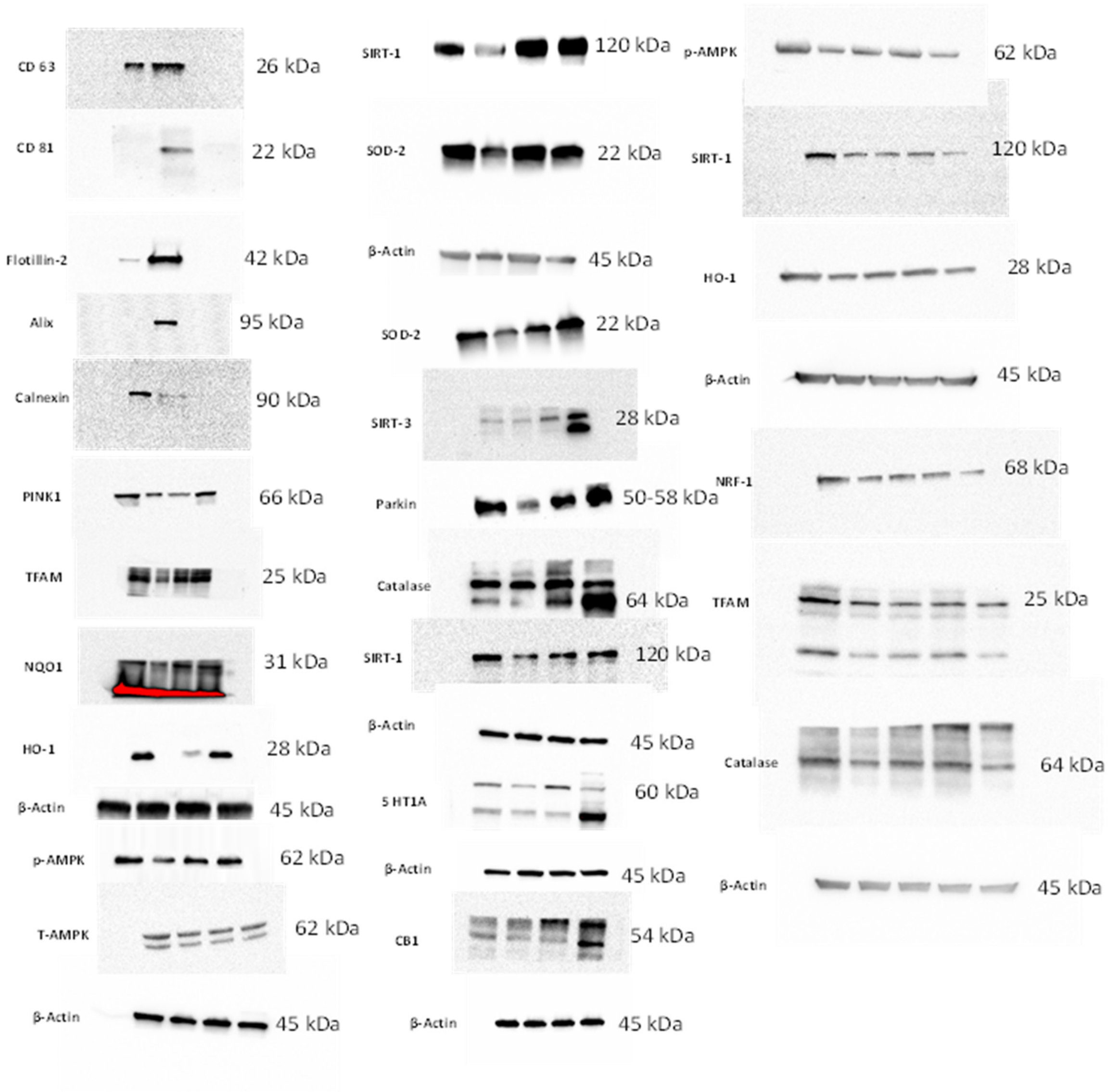

Supplement: Supplementary file 1 [file pharmaceutics-15-00554-s001.zip › pharmaceutics-2080797-supplementary.pdf]
